# Alpha oscillation mediates the interaction between suicide risk and symptom severity in Major Depressive Disorder

**DOI:** 10.3389/fnins.2024.1429019

**Published:** 2024-08-07

**Authors:** Haoran Zhang, Xinyu Liu, Ziyao Su, Yingtan Wang, Bingxu Chen, Zhizhen Zhang, Bin Wang, Jia Zhou, Ling Zhang, Xixi Zhao

**Affiliations:** ^1^Beijing Key Laboratory of Mental Disorders, National Clinical Research Center for Mental Disorders and National Center for Mental Disorders, Beijing Anding Hospital, Capital Medical University, Beijing, China; ^2^Advanced Innovation Center for Human Brain Protection, Capital Medical University, Beijing, China; ^3^Xinjiang Medical University, Urumqi, China; ^4^Faculty of Information Technology, Beijing University of Technology, Beijing, China; ^5^School of Mathematical Sciences, East China Normal University, Shanghai, China

**Keywords:** Major Depressive Disorder, electroencephalography, neural oscillation, suicide risk, intermediary effect

## Abstract

**Objective:**

The aim of our study was to explore the relationship between changes in neural oscillatory power in the EEG, the severity of depressive-anxiety symptoms, and the risk of suicide in MDD.

**Methods:**

350 MDD patients’ demographic and clinical data were collected, and their depressive and anxious symptoms were evaluated using HDRS-17 and HAMA-14, along with a suicide risk assessment using the Nurses’ Global Assessment of Suicide Risk (NGASR). EEG data were captured, processed, and analyzed to study brain activity patterns related to MDD. The participants were divided based on suicide risk levels, and statistical analyses, including chi-square, *t*-tests, Pearson’s correlations were used to explore the associations between brain activity, symptom severity, and suicide risk. Closely related variables were identified and ultimately the optimal model was screened using stepwise regression analysis with a forward strategy, and mediation effects were further used to determine the possible interactions between the variables in the regression model.

**Results:**

The regression model showed a significant effect of HDRS-17 and alpha power of Medial Occipital Cortex (MOC) on suicide risk, with elevated HDRS-17 increasing suicide risk and elevated alpha power decreasing suicide risk. Mediation effect analyses showed that MOC alpha power partially mediated the effect of depression level on suicide risk, and that an increase in depression severity may lead to a decrease in MOC alpha power, while a decrease in MOC alpha power may lead to an increase in suicide risk.

**Conclusion:**

The severity of depression directly increases suicide risk, whereas higher alpha power in the MOC serves as a protective factor, reducing this risk. Notably, MOC alpha power not only directly impacts suicide risk but also mediates the effects of both depression severity and anxiety levels on this risk.

**Limitations:**

The relatively small sample size of this study may limit the representativeness of the overall MDD patient population and the detailed analysis of different subgroups. This study did not delve into the relationship between the severity of cognitive symptoms in MDD patients and suicide risk.

## 1 Introduction

Major Depressive Disorder (MDD), a leading cause of disability worldwide, significantly impacts individuals’ lives through its profound effects on personal well-being, social interaction, and overall quality of life (Greenberg et al., 2021). A cohort study of 158,169 MDD reported 1.4% involved records of suicidal behavior. The all-cause mortality among patients with suicidal behavior was 2.6 times higher than among matched patients with MDD without records of suicidal behavior (Lundberg et al., 2023). The complex interplay between symptom severity and heightened suicide risk in Major Depressive Disorder (MDD) further complicates the clinical landscape. Early suicidal ideation and behavior in MDD are not always apparent in the primary complaint, posing certain difficulties for clinical identification and intervention (Núñez et al., 2020). Consequently, the search for biomarkers related to suicide risk is crucial for early identification and prevention of suicide in MDD.

Studies have shown that certain neurophysiological markers, including specific Electroencephalography (EEG) patterns, can predict suicidal ideation in patients with MDD, providing a potential avenue for identifying at-risk populations (de Aguiar Neto and Rosa, 2019). EEG facilitates the observation of subtle neural oscillation changes, providing insights into the disorder’s neurophysiological aspects (Yokoyama and Kitajo, 2022). Abnormal EEG neural oscillations may reflect an imbalance of excitation, inhibition, and hyperactivity in the cerebral cortex of MDD patients. Compared with healthy individuals, MDD showed significantly higher relative power of low delta and theta waves in the right occipital region and significantly lower relative power of α-waves throughout the posterior occipital region (Huang et al., 2023). Dolsen et al. (2017) found increased EEG fast-frequency activity, decreased delta activity, and increased alpha-delta sleep in participants with high suicidal ideation compared to those with low suicidal ideation. The study by Amico et al. investigated the EEG characteristics of suicidal ideation in depressed patients, with a particular focus on resting-state EEG manifestations, and they found that prefrontal EEG imbalances reflected higher anxiety and negative self-references but did not confirm the frequency-specific abnormalities proposed by previous studies in depressed patients (Amico et al., 2023). The study by Jiang et al. showed that decreased beta oscillations in MDD have a key role in promoting suicidal behavior, especially in those MDD patients who have recently attempted suicide, and decreased beta power is associated with increased suicidal behavior (Jiang et al., 2023).

However, current studies have mostly focused on the comparison of EEG characteristics between patients with high and low suicide risk MDD, and the lack of quantitative analyses between depression and anxiety levels, suicide risk and EEG characteristics may explain the heterogeneity between the results of those studies. Our study brings together a comprehensive set of data ranging from basic demographic details to clinical history and psychiatric assessment, combined with the neural oscillatory features of the EEG, with the aim of exploring the mediating role of EEG neural oscillatory features in MDD symptom severity and suicide risk.

## 2 Method

### 2.1 Participants

The study was conducted at Beijing Anding Hospital from July 2022 to May 2023. All patients receiving psychiatric services at the hospital during this period were consecutively invited to participate in the survey. The inclusion criteria consisted of age between 18 and 65 years, a diagnosis of MDD according to the Diagnostic and Statistical Manual of Mental Disorders, 5th edition (DSM-5) (Hasin et al., 2018) and a total score of ≥7 on the 17-item Hamilton Depression Rating Scale (HDRS-17). Exclusion criteria included the presence of a severe and unstable medical or surgical condition, a history of alcohol or substance abuse/dependence, and a diagnosis of dementia or other evident cognitive impairments. The study protocol received approval from the Ethics Committee of Beijing Anding Hospital (Registration Number: 2021–86), and all participants provided written informed consent following a thorough explanation of the study details. This study has completed clinical registration on https://www.chictr.org.cn/ (Clinical Trial Registration Number: ChiCTR2200059053).

### 2.2 Data collection and measurements

The primary socio-demographic and clinical data were collected using a form designed for this study. We collected the basic demographic information of participants, including gender, age, education level, marital status, history of alcohol use, history of tobacco use, family history of mental disorders, body mass index (BMI), number of MDD episodes, duration of MDD and current psychiatric medication. The severity of depressive and anxious symptoms was measured using the HDRS-17 (Hamilton Depression Rating Scale-17 items) and HAMA-14 (Hamilton Anxiety Scale-14 items). These scales are widely recognized in the mental health field for quantifying the severity of depression and anxiety disorders. The HDRS-17 is a 17-item scale that provides a comprehensive assessment of depressive symptomatology, with established validity and reliability in various populations (Kieslich da Silva et al., 2019). The HAMA-14, consisting of 14 items, is specifically designed to assess the severity of anxiety symptoms and has demonstrated sound psychometric properties (Maier et al., 1988). To evaluate suicide risk among MDD patients, we employed the Nurses’ Global Assessment of Suicide Risk (NGASR). The NGASR is a standardized assessment tool designed for healthcare practitioners to systematically evaluate and quantify suicide risk in patients. Its selection was based on its practicality in clinical settings and proven efficacy in predicting suicidal behaviors (Cutcliffe and Barker, 2004).

### 2.3 EEG signal collection and data processing

EEG signal acquisition was meticulously performed, capturing resting-state EEG data from participants through a structured protocol. All participants underwent a 10-min resting state recording with eyes closed and maintain a quiet, alert state. If a participant began to doze off, an auditory warning from the experimenter was issued. Any instances of warnings, opening eyes, or other non-resting states were marked and noted. Upon completion of the experiment, participants were assisted in washing off the conductive EEG paste from their scalp.

Data were obtained from 19 Ag/AgCl electrode channels using the advanced Neuracle system, which operates at a sampling rate of 1,000 Hz. The electrode cap, positioned according to the 10–20 system, utilized the default REF point as the reference electrode during EEG recordings, with impedance consistently kept below 5 kΩ. To control for potential distortion and fluctuations in both noise and signal, we implemented several measures: The EEG recording environment was carefully controlled for electrical and ambient noise. Participants were prepared adequately to minimize impedance, including skin preparation to reduce resistance. The Neuracle system was calibrated before each recording session to ensure optimal signal acquisition. Continuous monitoring of impedance levels was performed throughout the recording to detect and rectify any deviations promptly. Signal quality was assessed in real-time, with any segments affected by artifacts being marked for exclusion from subsequent analyses.

#### 2.3.1 EEG preprocessing

EEG data preprocessing utilized the EEGLAB toolbox within MATLAB R2013a for bandpass filtering (1–40 Hz), followed by down sampling to 500 Hz. The 10-min EEG dataset was initially segmented into consecutive 120-s intervals, with each of these intervals subsequently divided into 2-s segments. Two-second epochs were employed for artifact rejection and further analysis. Eye movement artifacts were removed by independent component analysis. Epochs with voltage excursions beyond ±150 μV were excluded. Subsequently, data were re-referenced to the average reference, and spectral power and asymmetry were computed for the 2-s epochs.

#### 2.3.2 Power spectrum

Power spectrum analysis was conducted using a Fast Fourier Transform (FFT) algorithm to quantify brain activity in the frequency domain, with power represented by the average instantaneous power of the analytic signal (Han et al., 2021a,b). Relative power for each frequency band was determined by normalizing the absolute power to the total broadband power (Liu et al., 2024), encompassing theta (4–8 Hz), alpha (8–12 Hz), beta1 (12–16 Hz), beta2 (16–24 Hz), and beta3 (24–40 Hz). Electrodes were categorized into 10 regions of interest (ROIs) for focused analysis: Prefrontal Cortex (PFC, including FP1, FP2, and Fz), Right Medial Frontal Cortex (RMFC, including Fz, F4, and F8), Left Medial Frontal Cortex (LMFC, including Fz, F3, and F7), Central Cortex (CC, including C3, C4, and Cz), Parietal Cortex (PP, including P3, P4, and Pz), Left Temporal Cortex (LT, including F7, T3, and T5), Right Temporal Cortex (RT, including F8, T4, and T6), Medial Occipital Cortex (MOC, including O1, Pz, and O2), Right Medial Occipital Cortex (RMOC, including P4, O2, and Pz), and Left Medial Occipital Cortex (LMOC, including P3, O1, and Pz).

### 2.4 Statistical analysis

Statistical analyses were performed with R version 4.0.3 and MATLAB 2013b. Based on the NGASR scores, we categorized the participants into a low to moderate suicide risk group (NGASR scores ≤8) and a high suicide risk group (NGASR scores ≥9) and made comparisons between the groups. Group comparisons for demographic and clinical variables were conducted using chi-square tests and t-tests, with a significance level set at *p* < 0.05 (two-tailed). Pearson’s correlations were used to examine the relationship between neural oscillatory characteristics, symptom severity, suicide risk, and general demographic characteristics. Step-wise linear regression analyses were used to explore independent variables that may have an effect on NGASR in the data of all participants. All variables significantly associated with NGASR were used as independent variables, and the best predictive model was found by adding or removing independent variables stepwise. A forward selection strategy was used to assess the goodness of the model based on the AIC (Akaike Informativeness Criterion) values. *p*-values for stepwise regression analysis and correlation analysis were corrected using False Discovery Rate (FDR). Ultimately, multiple regression analyses were used to explore direct and mediated effects between variables by constructing different regression models. Meanwhile, Bootstrap was used to estimate confidence intervals for the mediating effects to determine whether such effects are significant or not.

## 3 Results

### 3.1 Demographic and clinical characteristics of all participants

Data was gathered from 350 patients aged 18–65 (mean age 41.34 ± 14.864), with a gender split of 37.7% male and 62.3% female. The patients had an education range of 5–26 years (average 13.47 ± 3.562 years), and marital status was 32.9% single, 60.0% married, 4.0% divorced, and 3.1% widowed. BMI varied from 14.87 to 48.21 (average 23.93 ± 4.36). Alcohol use was reported by 2.9% and smoking by 9.7%. 76.6% had no family history of mental disorders. The number of depressive episodes was 1–21 (average 3.24 ± 2.80), with MDD duration of 0–40 years (average 7.10 ± 8.17 years). Scores on the HDRS-17 averaged 22.37 ± 5.50, on the HAMA averaged 19.61 ± 7.23, and on the NGASR, 7.05 ± 3.61. Antidepressant usage was 69.4%, antipsychotics 20.9%, mood stabilizers 7.1%, and anxiolytics 27.7%.

Utilizing the NGASR score, we stratified participants into two categories: a low-to-medium suicide risk group (LMS Group; NGASR score ≤ 8, *n* = 243) and a high suicide risk group (HS Group; NGASR score ≥ 9, *n* = 107), and between-group comparisons were made for all variables. NGASR scores were significantly lower in the LMS Group (5.24 ± 2.50) compared to the HS Group (11.18 ± 1.97; *t* = −23.856, *p* < 0.001). HDRS-17 scores were significantly lower in the LMS Group (21.49 ± 5.20) than in the HS Group (24.37 ± 5.66; *t* = −4.492, *p* < 0.001). Similarly, HAMA scores were significantly lower in the LMS Group (18.95 ± 7.21) compared to the HS Group (21.09 ± 7.11; *t* = −2.585, *p* = 0.010). Additionally, The LMS Group exhibited a notably lower BMI (23.52 ± 3.92) compared to the HS Group (24.86 ± 5.13; *t* = −2.368, *p* = 0.019). Antipsychotic medication use differed significantly between the LMS (17.7%) and HS (28.0%) Groups (*χ*^2^ = 4.813, *p* = 0.028). For other factors, including gender, age, education level, alcohol use, tobacco use, family history, marital status, number of episodes, duration of depression, antidepressant use, mood stabilizer use, anxiolytic use, and fluoxetine equivalence dose, there were no statistically significant differences between the two groups on these variables. The dose conversion formula for antidepressants is: fluoxetine 20 mg = citalopram 20 mg = escitalopram 9 mg = paroxetine 17 mg = sertraline 49.3 mg = venlafaxine 74.7 mg = mirtazapine 25.5 mg = agomelatine 26.6 mg = fluvoxamine 71.65 mg = duloxetine 20 mg (Table 1).

**Table 1:** Demographic and clinical characteristics of LMS and HS group.

Characteristics	LMS	HS	Statistics	
	(*n* = 243)	(*n* = 107)	*χ^2^/t*	*p* value
Female (%)	150 (61.7)	68 (63.6)	0.105	0.746
Age (years)^†^	41.73 ± 14.67	40.47 ± 15.33	0.718	0.473
Education level (years)^†^	13.62 ± 3.40	13.11 ± 3.91	1.110	0.269
Marital status (%)			1.857	0.603
Single	78 (32.1)	37 (34.6)		
Married	150 (61.7)	60 (56.1)		
Divorced	9 (3.7)	5 (4.7)		
Widowed	6 (2.5)	5 (4.7)		
BMI^†^	23.52 ± 3.92	24.86 ± 5.13	−2.368	0.019
Alcohol use (%)	8 (3.3)	2 (1.9)	0.542	0.462
Tobacco use (%)	23 (9.5)	11 (10.3)	0.056	0.812
Family history (%)	52 (21.4)	30 (28.0)	1.825	0.177
Number of episodes^†^	3.26 ± 2.91	3.20 ± 2.53	0.218	0.828
Duration of MDD (years)^†^	6.93 ± 8.13	7.47 ± 8.29	−0.558	0.578
Antidepressant use (%)	175 (72.0)	68 (63.6)	2.508	0.113
Fluoxetine equivalence dose (mg)^†^	40.84 ± 20.56	38.66 ± 19.56	0.767	0.444
Antipsychotic use (%)	43 (17.7)	30 (28.0)	4.813	0.028
Mood stabilizer use (%)	15 (6.2)	10 (9.3)	1.128	0.288
Anxiolytic use (%)	64 (26.3)	33 (30.8)	0.752	0.386
HDRS-17^†^	21.49 ± 5.20	24.37 ± 5.66	−4.492	<0.001
HAMA^†^	18.95 ± 7.21	21.09 ± 7.11	−2.585	0.010
NGASR^†^	5.24 ± 2.50	11.18 ± 1.97	−23.856	<0.001

^†^Mean ± SD; HDRS-17: the 17-item Hamilton Depression Rating Scale; HAMA, the Hamilton Anxiety Scale-14.

Separate between-group comparisons of the relative power of neural oscillations in each frequency band in whole brain regions of the two groups showed that the alpha power in the PFC (*t* = 2.23, *p* = 0.026), RMFC (*t* = 2.014, *p* = 0.045), LMFC (*t* = 1.997, *p* = 0.047), LT (*t* = 2.149, *p* = 0.032), MOC (*t* = 2.363, *p* = 0.019), RMOC (*t* = 2.158, *p* = 0.032), and LMOC (*t* = 2.307, *p* = 0.022) was significantly higher in the LMS Group compared to the HS Group. No significant group differences in relative power of oscillations in other frequency bands (Table 2).

**Table 2:** Comparison of relative power between in each frequency band in LMS and HS groups.

Characteristics	LMS (*n* = 243)	HS (*n* = 107)	Statistics	
	Mean ± SD	Mean ± SD	*t*	*p* value
RMOC_beta3	0.08 ± 0.06	0.09 ± 0.07	−1.706	0.090
LMOC_beta3	0.08 ± 0.06	0.09 ± 0.07	−1.499	0.136
MOC_beta3	0.08 ± 0.07	0.10 ± 0.08	−1.538	0.126
RT_beta3	0.11 ± 0.07	0.12 ± 0.08	−1.11	0.268
LT_beta3	0.12 ± 0.08	0.13 ± 0.09	−1.193	0.234
PP_beta3	0.07 ± 0.05	0.08 ± 0.07	−1.48	0.141
CC_beta3	0.12 ± 0.07	0.13 ± 0.08	−0.807	0.420
LMFC_beta3	0.12 ± 0.08	0.13 ± 0.09	−1.188	0.236
RMFC_beta3	0.12 ± 0.08	0.13 ± 0.09	−1.2	0.232
PFC_beta3	0.11 ± 0.08	0.13 ± 0.10	−1.589	0.114
RMOC_beta2	0.11 ± 0.06	0.12 ± 0.07	−1.293	0.198
LMOC_beta2	0.12 ± 0.06	0.13 ± 0.07	−1.351	0.178
MOC_beta2	0.11 ± 0.06	0.12 ± 0.07	−1.474	0.142
RT_beta2	0.14 ± 0.06	0.15 ± 0.07	−1.321	0.187
LT_beta2	0.14 ± 0.06	0.15 ± 0.07	−1.01	0.314
PP_beta2	0.12 ± 0.07	0.13 ± 0.07	−0.77	0.442
CC_beta2	0.15 ± 0.07	0.16 ± 0.08	−1.116	0.265
LMFC_beta2	0.14 ± 0.07	0.15 ± 0.08	−1.056	0.292
RMFC_beta2	0.14 ± 0.07	0.15 ± 0.08	−1.128	0.260
PFC_beta2	0.13 ± 0.07	0.14 ± 0.08	−0.987	0.325
RMOC_beta1	0.18 ± 0.08	0.18 ± 0.08	0.406	0.685
LMOC_beta1	0.18 ± 0.08	0.17 ± 0.08	0.171	0.865
MOC_beta1	0.17 ± 0.08	0.16 ± 0.07	0.251	0.802
RT_beta1	0.16 ± 0.07	0.16 ± 0.06	0.32	0.749
LT_beta1	0.15 ± 0.06	0.15 ± 0.06	0.598	0.550
PP_beta1	0.19 ± 0.08	0.19 ± 0.08	0.483	0.629
CC_beta1	0.17 ± 0.06	0.16 ± 0.06	0.472	0.638
LMFC_beta1	0.15 ± 0.06	0.14 ± 0.06	1.047	0.296
RMFC_beta1	0.15 ± 0.06	0.14 ± 0.06	1.02	0.308
PFC_beta1	0.15 ± 0.07	0.14 ± 0.06	1.04	0.299
RMOC_alpha	0.44 ± 0.14	0.41 ± 0.16	2.158	0.032
LMOC_alpha	0.44 ± 0.14	0.40 ± 0.15	2.307	0.022
MOC_alpha	0.44 ± 0.14	0.40 ± 0.15	2.363	0.019
RT_alpha	0.39 ± 0.13	0.36 ± 0.15	1.864	0.063
LT_alpha	0.38 ± 0.13	0.34 ± 0.14	2.149	0.032
PP_alpha	0.43 ± 0.14	0.40 ± 0.15	1.776	0.077
CC_alpha	0.36 ± 0.13	0.33 ± 0.14	1.895	0.059
LMFC_alpha	0.37 ± 0.14	0.34 ± 0.15	1.997	0.047
RMFC_alpha	0.37 ± 0.14	0.34 ± 0.15	2.014	0.045
PFC_alpha	0.38 ± 0.14	0.34 ± 0.16	2.23	0.026
RMOC_theta	0.18 ± 0.11	0.19 ± 0.10	−1.239	0.216
LMOC_theta	0.18 ± 0.11	0.20 ± 0.10	−1.319	0.188
MOC_theta	0.19 ± 0.11	0.21 ± 0.10	−1.362	0.174
RT_theta	0.19 ± 0.10	0.20 ± 0.10	−0.951	0.342
LT_theta	0.20 ± 0.10	0.22 ± 0.10	−1.331	0.184
PP_theta	0.18 ± 0.11	0.19 ± 0.10	−1.222	0.223
CC_theta	0.19 ± 0.10	0.20 ± 0.10	−1.32	0.188
LMFC_theta	0.21 ± 0.11	0.23 ± 0.10	−1.461	0.145
RMFC_theta	0.21 ± 0.11	0.23 ± 0.10	−1.394	0.164
PFC_theta	0.21 ± 0.11	0.23 ± 0.11	−1.527	0.128

PFC, Prefrontal Cortex; PP, Parietal Cortex; CC, Central Cortex; MOC, Medial Occipital Cortex; RMFC, Right Medial Frontal Cortex; LMFC, Left Medial Frontal Cortex; LT, Left Temporal Cortex; RT, Right Temporal Cortex; RMOC, Right Medial Occipital Cortex; LMOC, Left Medial Occipital Cortex.

### 3.2 Correlation analysis

**Figure 1:**
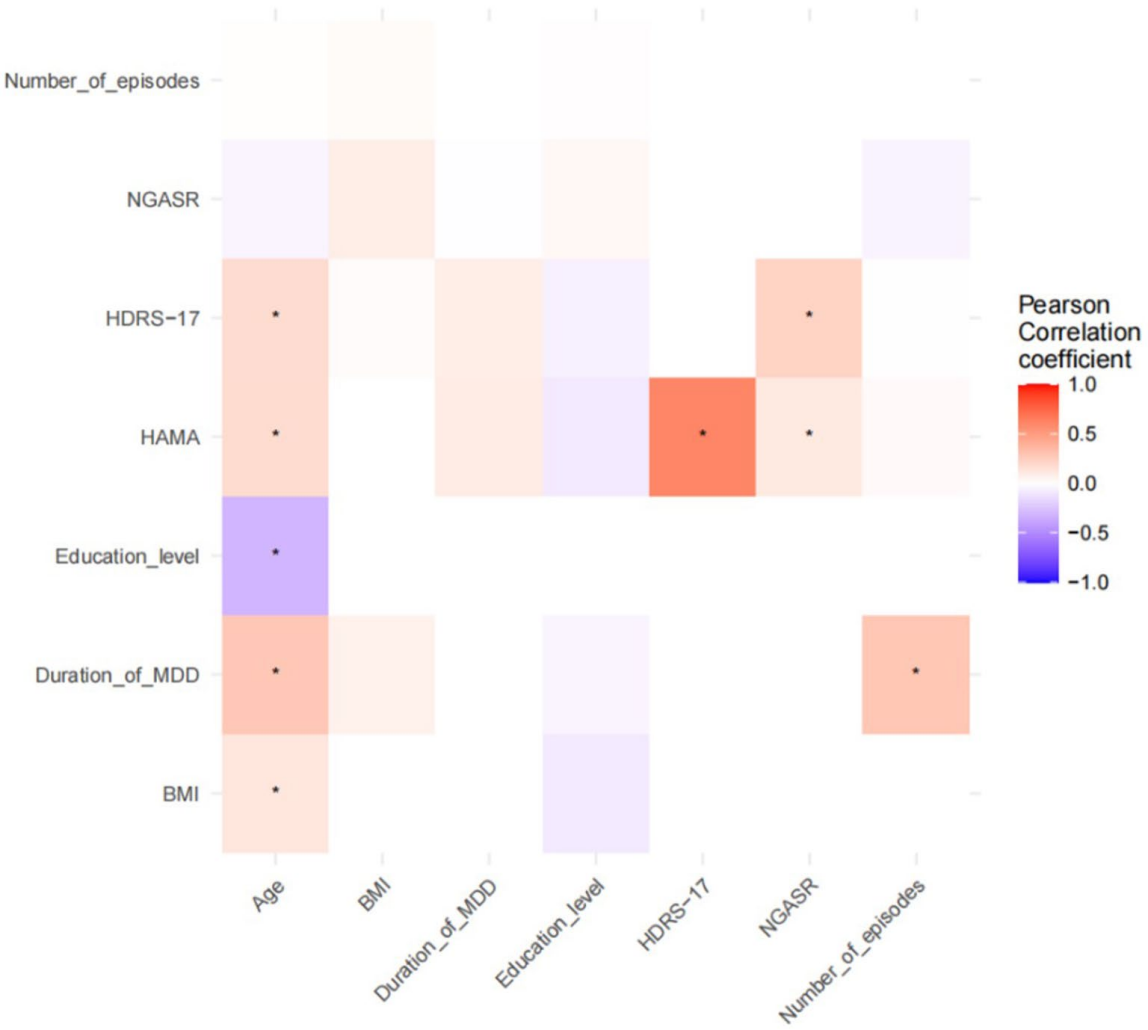
Pearson’s correlation analysis of demographic and clinical continuous variables. *p < 0.05.

**Figure 2:**
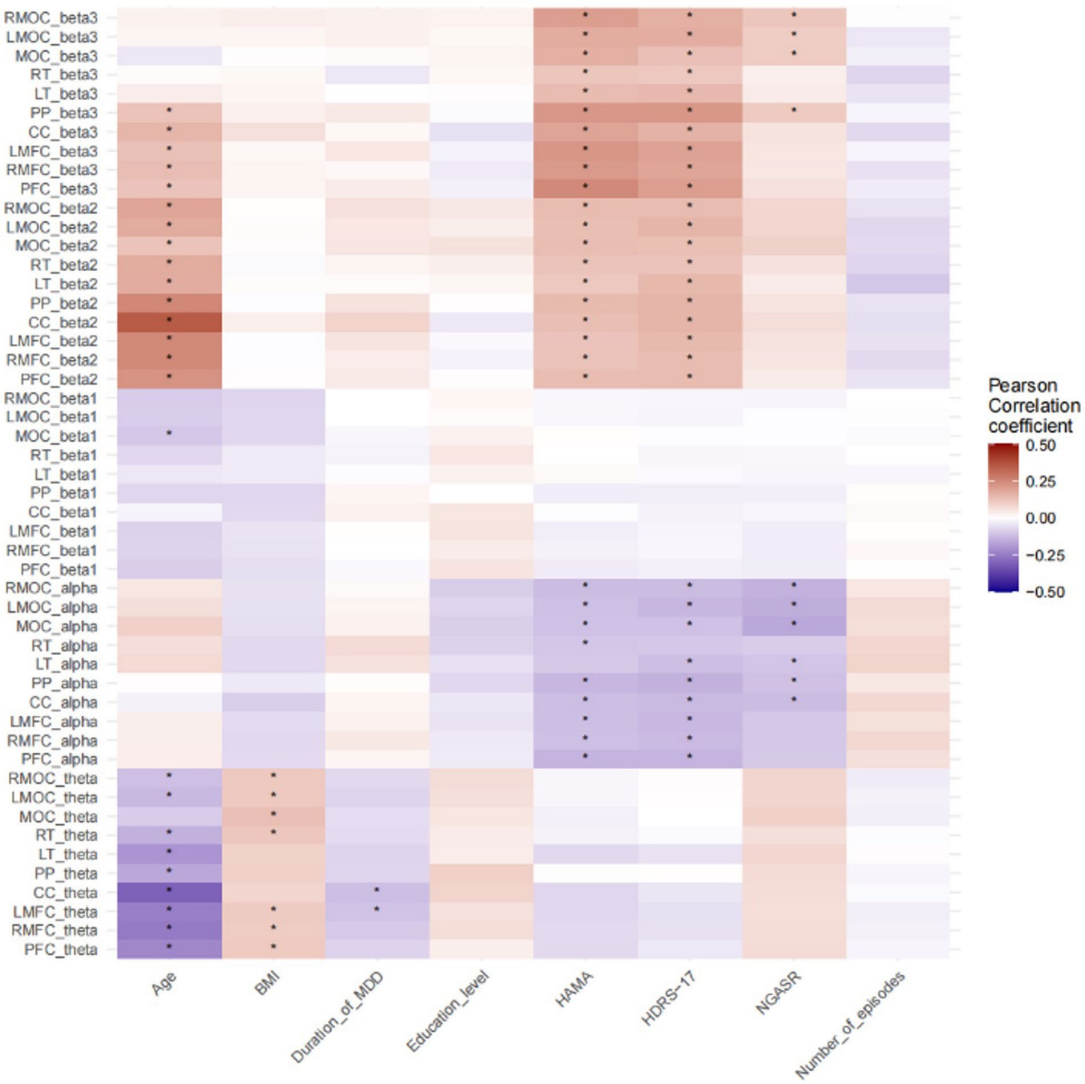
Pearson correlation analysis of demographic and clinical continuous variables with neural oscillatory power. *p < 0.05; PFC, Prefrontal Cortex; PP, Parietal Cortex; CC, Central Cortex; MOC, Medial Occipital Cortex; RMFC, Right Medial Frontal Cortex; LMFC, Left Medial Frontal Cortex; LT, Left Temporal Cortex; RT, Right Temporal Cortex; RMOC, Right Medial Occipital Cortex; LMOC, Left Medial Occipital Cortex.

Pearson correlation analyses of demographic information and clinical continuous variables with neural oscillatory power in each frequency band of each brain region were performed in all participants, and the results are shown in Figures 1, 2. NGASR was significantly positively correlated with HDRS-17 and HAMA, respectively (Figure 1). There was a significant positive correlation between HDRS-17 and HAMA, a significant positive correlation between age and BMI, disease duration, HDRS-17, HAMA, a significant negative correlation with education, and a significant positive correlation between disease duration and number of episodes (Figure 1).

NGASR showed negative correlation with alpha oscillatory power of RMOC, LMOC, MOC, LT, PP, and CC, and significant positive correlation with beta3 oscillatory power of RMOC, LMOC, MOC, and PP. HAMA scores were negatively correlated with alpha relative power in RMOC, LMOC,MOC, RT, PP, CC, LMFC, RMFC, PFC, positively correlated with beta3 relative power in RMOC, LMOC,MOC, RT LT, PP, CC, LMFC, RMFC, PFC, and positively correlated with RMOC, LMOC,MOC, RT, LT, PP, CC, LMFC, RMFC, PFC are positively correlated with beta2 relative power. HDRS-17 is negatively correlated with the alpha relative power of RMOC, LMOC, MOC, LT, PP,CC, LMFC, RMFC, PFC, positively correlated with the beta3 relative power of RMOC, LMOC, MOC, RT, LT, PP, CC, LMFC, RMFC, PFC, and positively correlated with the beta2 relative power of RMOC, LMOC, MOC, RT, LT, PP, CC, LMFC, RMFC, PFC LT, PP, CC, LMFC, RMFC, PFC. Age is negatively correlated with beta1 relative power for RMOC, LMOC, RT, LT, PP, CC, LMFC, RMFC, PFC, MOC and positively correlated with beta2 relative power for PP, CC, LMFC, RMFC, PFC, RMOC, LMOC, MOC, RT, LT, PP, CC, LMFC, RMFC, PFC, and BMI is positively correlated with theta relative power of RMOC, LMOC, MOC, RT, LMFC, RMFC, PFC (Figure 2). *p* values have been adjusted using the FDR correction.

### 3.3 Regression analysis

Stepwise linear regression analyses were used to explore independent variables that may have an effect on NGASR in the data of all participants. All variables significantly associated with NGASR were used as independent variables, and the best predictive model was found by adding or removing independent variables stepwise. A forward selection strategy was used to assess the goodness of the model based on the AIC (Akaike Informativeness Criterion) values. The final model contained three independent variables: HDRS-17, MOC alpha relative power, and LT alpha relative power. The results showed that the coefficient of HDRS-17 was 0.139 (*t* = 4.061, *p* < 0.001), indicating that HDRS-17 had a significant positive effect on NGASR. The coefficient of MOC alpha relative power was −6.77 (*t* = −2.636, *p* < 0.01), indicating that MOC alpha relative power had a significant negative effect on NGASR. The coefficient of LT alpha relative power was 4.30 (*t* = 1.495, *p* = 0.136), indicating a statistically insignificant effect of LT alpha relative power on NGASR. *p* values have been adjusted using the FDR correction.

### 3.4 Analysis of intermediation effects

**Figure 3:**
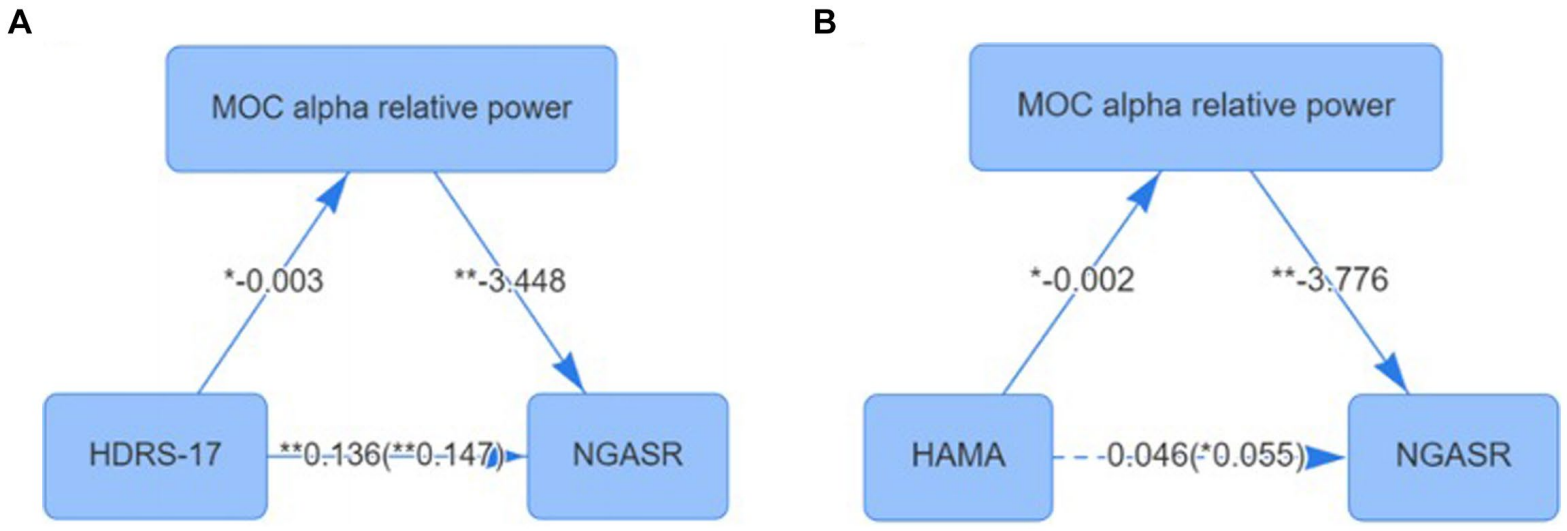
**(A)** Mediating effect of MOC alpha relative power between depression and suicide scores; **(B)** Mediating effect of MOC alpha relative power between anxiety Checked and suicide scores. *p < 0.05; **p < 0.01; MOC, Medial Occipital Cortex.

The NGASR score was used as the dependent variable, HDRS-17 as the independent variable, and MOC alpha relative power as the mediator variable, and the mediation effect model was constructed based on the percentile bootstrap method, and the results showed that the total effect value of the effect of HDRS-17 scores on the NGASR scores was 0.147 (*p* < 0.001), and the direct effect value was 0.136 (*p* < 0.001) the mediated effect value of MOC alpha relative power was 0.011 (95% BootCI: 0.001 ~ 0.037), with an effect share of 7.316%, and MOC alpha relative power partially mediated the effect of HDRS-17 on NGASR score (Figure 3A).

We further used NGASR score as the dependent variable, HAMA score as the independent variable, and MOC alpha relative power as the mediator variable for mediation effect model construction based on the percentile bootstrap method, and the results showed that the total effect value of the effect of HAMA on the NGASR score was 0.055 (*p* = 0.04), and the direct effect value was 0.046 (*p* = 0.084) the mediated effect value of MOC alpha relative power was 0.009 (95% BootCI: 0.002 ~ 0.042), with an effect share of 100%, and MOC alpha relative power played a fully mediated role in the effect of HAMA on NGASR scores (Figure 3B).

## 4 Discussion

This study is the first to quantitatively analyses the relationship between suicide risk, depression-anxiety level and EEG neural oscillation power in MDD patients. Our study indicated that higher severity of depression and anxiety is associated with lower alpha power in all brain regions, whereas all brain regions show higher beta2 and beta3 relative power. Suicide risk is negatively correlated with alpha power in multiple brain regions, and positively correlated with beta3 power in specific brain regions. Regression analysis identified HDRS-17 score and MOC alpha relative power as predictors of suicide risk. Mediation effect analyses showed that MOC alpha power partially mediated the effect of depression level on suicide risk, and that increased depression severity may lead to decreased MOC alpha power, while decreased MOC alpha power may lead to increased suicide risk. MOC alpha power fully mediated the effect of HAMA on suicide risk, the magnitude of this effect was small but statistically significant, and an increase in anxiety may lead to a decrease in MOC alpha power, which may lead to an increase in suicide risk.

The results of the between-group comparisons showed that the severity of depression and anxiety was significantly higher in MDD patients at high suicide risk than in those at low to moderate suicide risk, which is consistent with the results of previous studies (Li et al., 2021). The notably lower alpha oscillation relative power in specific brain regions, including the PFC, LT, and various motor cortex regions (MOC, RMOC, LMOC), in the HS Group compared to the LMS Group is consistent with previous research linking alpha power to the severity of depression and anxiety symptoms (Jiang et al., 2016). Meanwhile, the proportion of antipsychotic use in the medication regimen of depressed patients with high suicide risk was greater than that of those with low to moderate suicide risk, which is closely related to clinical decision-making based on evidence-based medicine (Ruengorn et al., 2012; Nuñez et al., 2022). Many studies have shown an inverse relationship between BMI and suicide, meaning that obese people are less likely to commit suicide than people of low or normal weight (Perera et al., 2016). However, in the sample included in this study, BMI was higher in those with high suicide risk than those with low to moderate suicide risk. This result might be related to the high rate of antipsychotic use in depressed patients at high risk of suicide, which highlighted the intricate relationship between medication regimens, BMI, and suicide risk in MDD patients, emphasizing the need for comprehensive considerations of clinical and pharmacological factors in assessing suicide risk in this population (Hecht et al., 2021).

Based on the correlation analysis results, it is evident that there are significant associations between EEG spectral power, demographic and clinical variables, as well as symptom severity and suicide risk in MDD patients. The positive correlation between NGASR and HDRS-17 as well as HAMA scores underscores the relationship between suicide risk and symptom severity in MDD patients. This finding aligns with previous research indicating that higher depression and anxiety scores are associated with increased suicide risk in individuals with MDD (Conejero et al., 2018; Stanley et al., 2018). Depression and anxiety severity showed a positive correlation with beta2 and beta3 relative power in the whole brain, and a negative correlation with alpha relative power in almost all brain areas. This is consistent with previous studies. Chang and Choi showed that decreases in Alpha/Beta power Ratios (ABR) in central brain areas resulted in increased scores on the Beck Depression Inventory and Spielberg Trait Anxiety Inventory, and that global ABR of the brain are a potentially objective indicator for diagnosing MDD (Chang and Choi, 2023). The overall decrease in ABR may be related to broader changes in functional connectivity during the depressive resting state (Mulders et al., 2015; Helm et al., 2018). Interestingly, NSGAR scores and depression-anxiety severity showed a similar trend of association with alpha and beta oscillatory power, the negative correlation between NGASR and alpha oscillatory power in several brain regions, coupled with the positive correlation with beta3 oscillatory power, which aligns with existing literature suggesting aberrant alpha and beta oscillatory patterns in MDD patients (Dai et al., 2022). It has been suggested that reduced beta1 power moderates the transition from suicidal ideation to suicide in MDD, which may increase the risk of suicide, and the trend of the results of the present study is in line with that (Jiang et al., 2023).

Furthermore, the associations between demographic variables such as age, education, and BMI with specific EEG frequency bands highlight the multifaceted nature of neural oscillatory dynamics in MDD. For instance, the negative correlation between age and beta1 power, and the positive correlation with beta2 power, underscores the impact of age-related changes on neural oscillations, which has been previously documented in the literature (Smith et al., 2023). Similarly, the positive correlation between BMI and theta power aligns with studies linking obesity and altered neural oscillations, indicative of potential neurobiological mechanisms underlying the association between obesity and MDD (Jáuregui-Lobera, 2011).

The results of the regression analysis in our study revealed several significant findings regarding the relationship between EEG spectral power and suicide risk in MDD patients. Specifically, the stepwise linear regression analyses identified HDRS-17 score and MOC alpha relative power as the best predictive model for NGASR. The coefficient of HDRS-17 demonstrated a significant positive effect on NGASR, indicating that depress severe were associated with increased suicide risk. This aligns with previous research highlighting the strong correlation between depressive symptom severity and suicide risk in MDD patients (Martinengo et al., 2019). Furthermore, the relative power of MOC alpha showed a significant negative effect on NGASR, suggesting that lower MOC alpha relative power was linked to elevated suicide risk. This finding is consistent with the notion that aberrant alpha power may reflect disrupted neural activity underlying emotional dysfunctions in MDD, thus contributing to heightened suicide risk (Umemoto et al., 2021).

A quantitative resting EEG study of Hispanic female adolescents with suicidal ideation and matched normal controls showed that the right hemisphere of normal adolescents had greater alpha (lower activation) than the left hemisphere, whereas suicidal adolescents did not have significant asymmetry in the opposite direction. Alpha asymmetry in the posterior region was associated with the score of suicidal intent, but not with depression severity (Graae et al., 1996), our findings add to those of previous studies. Moreover, our analysis of intermediation effects shed light on the mediating role of MOC alpha relative power in the relationship between depressive symptoms and suicide risk. The mediation effect model indicated that MOC alpha relative power partially mediated the effect of HDRS-17 on NGASR scores, with an effect share of 7.316%. This underscores the potential of MOC alpha relative power as a neurophysiological marker that partially explains the link between depressive symptomatology and suicide risk in MDD patients (Dai et al., 2022). Similarly, the mediation effect model involving the HAMA scores revealed that MOC alpha relative power played a fully mediated role in the effect of HAMA on NGASR scores. This suggests that MOC alpha relative power may serve as a comprehensive indicator of suicide risk, encompassing both depressive and anxiety-related pathways. For instance, a study by Ballard et al. (2022) demonstrated a significant association between decreased alpha power and increased suicidal behaviors in individuals with MDD, highlighting the potential utility of alpha power as a neurophysiological marker for suicide risk assessment.

When discussing the limitations of this study, it is important to acknowledge certain factors that may impact the interpretation and generalizability of the findings. Firstly, the relatively small sample size of this study may limit the representativeness of the overall MDD patient population and the detailed analysis of different subgroups. Larger-scale studies may provide a more comprehensive understanding of the relationship between EEG spectral power dynamics and suicide risk in MDD patients. Secondly, this study did not delve into the relationship between the severity of cognitive symptoms in MDD patients and suicide risk. Cognitive symptoms may play a significant role in MDD patients and could be associated with EEG spectral dynamics (Wang et al., 2024). Therefore, the lack of comprehensive consideration of cognitive symptoms may limit a comprehensive understanding of factors related to suicide risk in MDD patients.

## 5 Conclusion

The study conclusively demonstrates that among patients with MDD, the severity of depression directly increases suicide risk, whereas higher alpha power in the MOC serves as a protective factor, reducing this risk. The absence of the HAMA in the final regression model suggests a complex interplay of factors influencing suicide risk, potentially moderated by covariance among variables. Notably, MOC alpha power not only directly impacts suicide risk but also mediates the effects of both depression severity and anxiety levels on this risk. This highlights the pivotal role of brain activity patterns, particularly in the MOC, in modulating the relationship between psychological symptoms and the propensity for suicide in individuals with MDD.

## Data availability statement

The raw data supporting the conclusions of this article will be made available by the authors, without undue reservation.

## Ethics statement

The studies involving humans were approved by the Ethics Committee of Beijing Anding Hospital. The studies were conducted in accordance with the local legislation and institutional requirements. The participants provided their written informed consent to participate in this study.

## Author contributions

HZ: Data curation, Formal analysis, Investigation, Writing – original draft. XL: Data curation, Formal analysis, Writing – original draft. ZS: Investigation, Methodology, Software, Supervision, Validation, Writing – review & editing. YW: Data curation, Methodology, Visualization, Writing – review & editing. BC: Methodology, Software, Visualization, Writing – review & editing. ZZ: Project administration, Resources, Validation, Visualization, Writing – review & editing. BW: Data curation, Project administration, Resources, Software, Visualization, Writing – review & editing. JZ: Formal analysis, Investigation, Project administration, Resources, Writing – review & editing. LZ: Project administration, Supervision, Writing – review & editing. XZ: Conceptualization, Methodology, Resources, Writing – review & editing.
